# Stochastic Comparisons of Weighted Distributions and Their Mixtures

**DOI:** 10.3390/e22080843

**Published:** 2020-07-30

**Authors:** Abdulhakim A. Albabtain, Mansour Shrahili, M. A. Al-Shehri, M. Kayid

**Affiliations:** 1 Department of Statistics and Operations Research College of Science, King Saud University, Riyadh 11451, Saudi Arabia; hakim@ksu.edu.sa (A.A.A.); msharahili@ksu.edu.sa (M.S.); mashael@ksu.edu.sa (M.A.A.-S.); 2 Department of Mathematics and Computer Science, Faculty of Science, Suez University, Suez 14028, Egypt

**Keywords:** stochastic order, mixture distribution, hazard rate, preservation, coherent systems, shannon entropy

## Abstract

In this paper, various stochastic ordering properties of a parametric family of weighted distributions and the associated mixture model are developed. The effect of stochastic variation of the output random variable with respect to the parameter and/or the underlying random variable is specifically investigated. Special weighted distributions are considered to scrutinize the consistency as well as the usefulness of the results. Stochastic comparisons of coherent systems made of identical but dependent components are made and also a result for comparison of Shannon entropies of weighted distributions is developed.

## 1. Introduction

In the literature, weighted distributions have been exhaustively applied and put to use to model data in nature, as they provide more insights to provide more adequacy in modelling as a result of variety of sampling surveys (cf. Rao [1], Patil and Rao [2] and Patil [3]). Let *X* be a random variable with cumulative distribution function (cdf) *F* and probability density function (pdf) *f* and let w(·,θ) is a non-negative function such that E(w(X,θ)) exists and is finite for all θ∈χ, where χ is an arbitrary subset of R. Then $X_{w}$ is taken to be a random variable with weighted distribution associated with *f*, having pdf

(1) $$f_{w}\left(x,\theta \right)=\frac{w(x,\theta )f(x)}{E[w(X,\theta )]}.$$

Many families of statistical distributions hold at the disposal of the family of weighted distributions in (1) (see, e.g., the typical weighted distributions in Section 3.1 and Section 3.2). Suppose that the hazard rate function *h* corresponds to the pdf *f* so that $h\left(x\right)=f\left(x\right)/\bar{F}\left(x\right)$ where $\bar{F}\equiv 1-F$ is the survival function of *X*. In spirit of Jain et al. [4], the hazard rate of $X_{w}$ is characterized by

(2) $$h_{w}\left(x,\theta \right)=\frac{w(x,\theta )}{B(x,\theta )}h\left(x\right),$$

in which B(x,θ)=E[w(X,θ)∣X>x]. As for the reversed hazard rate of $X_{w}$ we have from Sunoj and Maya [5]

(3) $${\tilde{h}}_{w}\left(x,\theta \right)=\frac{w(x,\theta )}{A(x,\theta )}\tilde{h}\left(x\right),$$

where A(x,θ)=E[w(X,θ)∣X≤x] and $\tilde{h}\left(x\right)=f\left(x\right)/F\left(x\right)$ is the reversed hazard rate function of *X*.

The density in (1) may be used to model data randomly drawn from population at a certain level θ of some quantity of interest. For example, θ could be a particular age for an individual, a certain time point or a given threshold with a specific amount. In many realistic circumstances it is acknowledged that the parameter θ may not be constant so that the occurrence of heterogeneity is sometimes incalculable and unexplained. In addition, it often takes place in practical situations where data from several populations is mixed. To model such data sets mixture models are used. For example, the measurements on the life lengths of a device may be gathered regardless of the manufacturer, or data may be gathered on humans without regard, say, to blood type. If the ignored variable has a bearing on the characteristic which is being measured, then the data follow a mixture model. To all intents and purposes, it is hard to find data that are not some kind of a mixture, because there is almost always some relevant covariate that is not observed.

The study of reliability properties of various mixture models has recently received much attention in the literature. When a mixture model is fitted to survival data, the mixing operation can change the pattern of aging for the lifetime unit under consideration in some favorite way (see, for example, Finkelstein and Esaulova [6], Alves and Dias [7], Arbel et al. [8], Cole and Bauer [9], Bordes and Chauveau [10], Li and Liu [11], Amini-Seresht and Zhang [12], Misra and Naqvi [13] and Badía and Lee [14]).

Mixture models capture heterogeneity in data by decomposing the population into latent subgroups, each of which is governed by its own subgroup-specific set of parameters. To represent a general formulation of the mixture model in the case of our study, consider the density

(4) $$f^{*}\left(x\right)=\int_{\theta \in \chi }f_{w}\left(x,\theta \right)\;dG\left(\theta \right)=f\left(x\right)\int_{\theta \in \chi }\frac{w(x,\theta )}{\mu (\theta )}\;dG\left(\theta \right),$$

associated with (1), where μ(θ)=E(w(X,θ)) and *G* is the cdf of the random varaible Θ. It is known that $f^{*}\left(x\right)=f\left(x\right)v\left(x\right)$ with

(5) $$v\left(x\right)=E\left(\frac{w(x,\Theta )}{\mu (\Theta )}\right),$$

playing the role of the weight function through which *f* is altered to $f^{*}.$ This signifies that the mixture density in (4) can be thought as the density of a weighted distribution with weight function *v* for which E(v(X))=1. In situations where Θ is designated by a discrete random variable, a finite mixture model is considered. To this end, the model (4) is developed as

(6) $$f^{*}\left(x\right)=f\left(x\right)\sum_{i=1}^{\infty }\frac{w(x,\theta_{i})}{\mu (\theta_{i})}g\left(\theta_{i}\right),$$

where $g(\theta_{i})$ represents the value of the probability mass function (pmf) of Θ at $\theta_{i}$ for i=1,2,⋯. Throughout the paper, it is assumed that the output random variables following the mixture weighted distribution (4) have absolutely continuous distribution functions.

To the best of our knowledge, there has not been a work on the literature to argue different stochastic properties of the parametric weighted distributions as well as their mixtures in general to be attractive for broader audiences. There is a need for an effective study in this direction. The main objective of this paper is to initiate such a study to investigate the impact of the association of the model to a parameter on some general stochastic aspects of the model.

The rest of the paper is organized as follows. In Section 2, some useful notions of stochastic orders and some further stochastic properties are presented. In Section 3, some special applied weighted distributions are introduced. In Section 4, preservation of several ordinary as well as relative stochastic orderings is studied in Section 4.1. In Section 4.2, preservation properties of some stochastic orders in the extended mixture model of weighted distributions are secured and in the long run in Section 4.3, a link to information theory is provided.

## 2. Preliminaries

Assume that the random variables *X* and *Y* have distribution functions *F* and *G*, survival functions $\bar{F}=1-F$ and $\bar{G}=1-G$, density functions *f* and *g*, hazard rate functions $h_{X}=f/\bar{F}$ and $h_{Y}=g/\bar{G}$ and reversed hazard rate functions ${\tilde{h}}_{X}=f/F$ and ${\tilde{h}}_{Y}=g/G$, respectively. To compare the magnitude of random variables some notions of stochastic orders are introduced below.

**Definition** **1.** *The random variable X is said to be smaller than the random variable Y in the*

*(i)*  *usual stochastic order (denoted by $X\leq_{st}Y$) if $\bar{F}\left(x\right)\leq \bar{G}\left(x\right)$ for all x;*
*(ii)*  *hazard rate order (denoted by $X\leq_{hr}Y$) if $\bar{G}\left(x\right)/\bar{F}\left(x\right)$ is non-decreasing in x, or equivalently, if $h_{X}\left(x\right)\geq h_{Y}\left(x\right)$ for all x;*
*(iii)*  *reversed hazard rate order (denoted by $X\leq_{rh}Y$) if G(x)/F(x) is non-decreasing in x, or equivalently, if ${\tilde{h}}_{X}\left(x\right)\leq {\tilde{h}}_{Y}\left(x\right)$ for all x;*
*(iv)*  *likelihood ratio order (denoted by $X\leq_{lr}Y$) if g(x)/f(x) is non-decreasing in x.*
*(v)*  *relative hazard rate order (denoted by $X\leq_{rhr}Y$) if $h_{Y}\left(x\right)/h_{X}\left(x\right)$ is non-increasing in x.*
*(vi)*  *relative reversed hazard rate order (denoted by $X\leq_{rrh}Y$) whenever ${\tilde{h}}_{Y}\left(x\right)/{\tilde{h}}_{X}\left(x\right)$ is non-decreasing in x.*

It is known that the following implications hold:

$$X\leq_{lr}Y\;\Rightarrow \;X\leq_{hr[rh]}Y\;\Rightarrow \;X\leq_{st}Y.$$

The notions of the totally positive of order 2 (TP2) and the reverse regular of order 2 (RR2) are defined as follows.

**Definition** **2.** (Karlin [15]). *A function h(x,y) is said to be Sign-Regular of order 2 (SR${}_{2}$) if $\epsilon_{1}h\left(x,y\right)\geq 0$ and*

$$\epsilon_{2}\left|\begin{array}{cc} h(x_{1},y_{1}) & h(x_{1},y_{2})\\ h(x_{2},y_{1}) & h(x_{2},y_{2}) \end{array}\right|\geq 0,$$

*for all $x_{1}\leq x_{2}$ and for all $y_{1}\leq y_{2}$ for $\epsilon_{1}$ and $\epsilon_{2}$ equaling to +1 or −1.*

If $\epsilon_{1}=+1$ and $\epsilon_{2}=+1$, then *h* is said to be TP${}_{2}$. If $\epsilon_{1}=+1$ and $\epsilon_{2}=-1$, then *h* is said to be RR${}_{2}$. It is readily pointed out that the TP${}_{2}$ [RR${}_{2}$] property of h(t,x) is equivalent to saying that $h\left(t,x_{2}\right)/h\left(t,x_{1}\right)$ is non-decreasing [non-increasing] in *t* whenever $x_{1}\leq x_{2}$ after making the conventions that a/0=+∞ when a>0 and a/0=0 if a=0. In view of the foregoing statements and by assuming $h_{Y}=h_{2}$ and $h_{X}=h_{1}$ and also ${\tilde{h}}_{Y}={\tilde{h}}_{2}$ and ${\tilde{h}}_{X}={\tilde{h}}_{1}$, one observes $X_{1}\leq_{rhr}X_{2}$ holds if, and only if, $h_{i}\left(x\right)$ is RR${}_{2}$ as a function of (i,x)∈{1,2}×ζ, where ζ is the common support of *X* and *Y*. In a similar manner we can establish that $X_{1}\leq_{rrh}X_{2}$ is equivalent to ${\tilde{h}}_{i}\left(x\right)$ being TP${}_{2}$ in (i,x)∈{1,2}×ζ.

## 3. Special Weighted Distributions

In this section, several special parametric weight functions are presented making the investigation of the main model of (4) more developed. First, some general formations of the weight function are considered by which many important families of weighted distributions are included. In all of the cases we assume that the weight function has a finite mean with respect to the underlying distribution.

### 3.1. Distribution-Free Weight Functions

Here, several weight functions which do not depend on the underlying distribution are given. Suppose that $w_{i},i=1,2$ are two non-negative functions of *x* and that $k_{i},i=1,2$ are two proper functions of θ so that the following weight functions satisfy the requirement that μ(θ)<∞. Substituting any of these weight functions in the density (4) leads to a particular model that might be of some interest in a context.

(i) (weighted power) $w\left(x,\theta \right)=w_{1}\left(x\right){\left(w_{2}\left(x\right)\right)}^{k_{1}\left(\theta \right)}$
(ii) (weighted exponentiated) $w\left(x,\theta \right)=w_{1}\left(x\right){\left(k_{1}\left(\theta \right)\right)}^{w_{2}\left(x\right)}$
(iii) (multiplicative) $w\left(x,\theta \right)=w_{1}\left(x\right)k_{1}\left(\theta \right)$
(iv) (additive-multiplicative) $w\left(x,\theta \right)=w_{1}\left(x\right)+k_{1}\left(\theta \right)w_{2}\left(x\right)$
(v) (weighted left-truncated) $w\left(x,\theta \right)=w_{1}\left(x\right)I\left[w_{2}\left(x\right)>k_{1}\left(\theta \right)\right]$
(vi) (weighted right-truncated) $w\left(x,\theta \right)=w_{1}\left(x\right)I\left[w_{2}\left(x\right)\leq k_{1}\left(\theta \right)\right]$

### 3.2. Semiparametric Models

Models where the parameters of interest are finite-dimensional and the nuisance parameters are infinite-dimensional are called semiparametric models. There are some choices for the weight function w(x,θ) that are functional of the underlying distribution function F, including the parameter θ within. Below, we list some kinds of those choices whose associated weight function depend on the underlying distribution.

(i) (Proportional hazards) $w\left(x,\theta \right)={\bar{F}}^{\theta -1}\left(x\right),$ where θ>0.
(ii) (Proportional reversed hazards) $w\left(x,\theta \right)=F^{\theta -1}\left(x\right)$ where θ>0.
(iii) (Proportional odds ratio) $w\left(x,\theta \right)=1/({\left(1-\bar{\theta }\bar{F}\left(x\right)\right)}^{2})$ where θ>0, and $\bar{\theta }=1-\theta .$
(iv) (Upper records) $w\left(x,\theta \right)=-\ln^{\theta }\left(\bar{F}\left(x\right)\right),$ in which θ∈N.
(v) (Lower records) $w\left(x,\theta \right)=-\ln^{\theta }\left(F\left(x\right)\right),$ in which θ∈N.
(vi) (Residual life) w(x,θ)=f(θ+x)/f(x), where θ>0 is the guaranteed survival time.
(viii) (Inactivity time) w(x,θ)=f(θ−x)/f(x), in which θ>0 is the time of observation of failure.
(viii) (Scale) w(x,θ)=f(θx)/f(x), in which θ>0.

## 4. Stochastic Orderings

In this section, preservation properties of some stochastic orders under the formation of the weighted model in the fixed as well as the random levels of the parameter θ are studied.

### 4.1. Weighted Distribution with Specific Parameter

Here, in the same vein as Misra et al. [16] several preservation properties on likelihood ratio, hazard rate and reversed hazard rates orders can be established in the sense of the model (1). Suppose that $X_{i}$ is a random variable with pdf $f_{i}$ and cdf $F_{i},$ for i=1,2, and assume that $X_{iw_{i}}$ follows the weighted distribution of $X_{i}$ with weight function $w_{i}\left(x\right)=w\left(x,\theta_{i}\right)$ having pdf

(7) $$f_{iw_{i}}\left(x,\theta_{i}\right)=\frac{w\left(x,\theta_{i}\right)f_{i}\left(x\right)}{E[w\left(X_{i},\theta_{i}\right)]},\;i=1,2,$$

where $\theta_{1}$ and $\theta_{2}$ are two fixed numbers in χ. In the next round, as will be presented, conditions for stochastic orders made of $X_{1w_{1}}$ and $X_{2w_{2}}$ to emulate the same type of stochastic orders between $X_{1}$ and $X_{2}$ are obtained.

The following Proposition is a direct conclusion of Theorem 3.2 in Misra et al. [16].

**Proposition** **1.** *Let $X_{i},i=1,2$ have support ζ and let w(x,θ) be TP${}_{2}$ in (x,θ)∈ζ×χ. Then*

*(i)*  *If $\theta_{1}\leq \theta_{2},$ then $X_{1}\leq_{lr}X_{2}$ implies that $X_{1w_{1}}\leq_{lr}X_{2w_{2}}.$*
*(ii)*  *If $\theta_{1}\leq \theta_{2}$ and w(x,θ) is non-decreasing in x, then $X_{1}\leq_{hr}X_{2}$ implies that $X_{1w_{1}}\leq_{hr}X_{2w_{2}}.$*
*(iii)*  *If $\theta_{1}\leq \theta_{2}$ and w(x,θ) is non-increasing in x, then $X_{1}\leq_{rh}X_{2}$ implies that $X_{1w_{1}}\leq_{rh}X_{2w_{2}}.$*

Preservation properties of the stochastic orders considered in Proposition 1 have been procured for some special weighted distributions by Izadkhah et al. [17] including the models of proportional (reversed) hazard rates, upper (lower) records, right (left) truncation, moment generating and size-biased distributions. Izadkhah et al. [18] obtained sufficient conditions for preservation of reversed mean residual life order and Izadkhah et al. [19] presented some conditions under which the mean residual life order is preserved under weighting. For the sake of completeness, the preservation properties of the likelihood ratio, the hazard rate and the reversed hazard rates orders are studied for some of the parametric weighted distributions considered in Section 3.1 and Section 3.2. Suppose that $X_{1}$ and $X_{2}$ are two non-negative random variables with distribution functions $F_{1}$ and $F_{2}$, survival functions ${\bar{F}}_{1}=1-F_{1}$ and ${\bar{F}}_{2}=1-F_{2}$ and density functions $f_{1}$ and $f_{2}$, respectively.

**Example** **1** (Weighted power distribution)**.** *Assume that*

$$w\left(x,\theta_{i}\right)=w_{1}\left(x\right){\left(w_{2}\left(x\right)\right)}^{k_{1}\left(\theta_{i}\right)},i=1,2,$$

*in which $\theta_{1}\leq \theta_{2}\in \chi$. Suppose that $w_{2}$ and $k_{1}$ are both non-decreasing (or non-increasing) functions. By Proposition 1(i), if $X_{1}\leq_{lr}X_{2}$ then $X_{1w_{1}}\leq_{lr}X_{2w_{2}}.$ Let us further assume that $k_{1}\left(\theta_{i}\right)>0$ for i=1,2. Then, by Proposition 1(ii) $X_{1}\leq_{hr}X_{2}$ implies $X_{1w_{1}}\leq_{hr}X_{2w_{2}}$ provided that $w_{1}$ and $w_{2}$ are both non-decreasing. In parallel, if $w_{1}$ and $w_{2}$ are both non-increasing then using Proposition 1(iii), $X_{1}\leq_{rh}X_{2}$ concludes that $X_{1w_{1}}\leq_{rh}X_{2w_{2}}$.*

**Example** **2** (Weighted exponentiated distribution)**.** *Consider the weight function*

$$w\left(x,\theta_{i}\right)=w_{1}\left(x\right){\left(k_{1}\left(\theta_{i}\right)\right)}^{w_{2}\left(x\right)},i=1,2$$

*such that $\theta_{1}\leq \theta_{2}\in \chi$ in which $k_{1}\left(\theta_{i}\right)>0$ for i=1,2. Presume that $w_{2}$ and $k_{1}$ are both non-decreasing (or non-increasing) functions. From Proposition 1(i), $X_{1}\leq_{lr}X_{2}$ yields $X_{1w_{1}}\leq_{lr}X_{2w_{2}}.$ If $w_{1}$ is non-decreasing, $w_{2}$ is non-decreasing and $k_{1}\left(\theta_{i}\right)>1$ (resp. $w_{2}$ is non-increasing and $k_{1}\left(\theta_{i}\right)<1$) for i=1,2 then by Proposition 1(ii) $X_{1}\leq_{hr}X_{2}$ gives $X_{1w_{1}}\leq_{hr}X_{2w_{2}}$. Besides, if $w_{1}$ is non-increasing, $w_{2}$ is non-increasing and $k_{1}\left(\theta_{i}\right)>1$ (resp. $w_{2}$ is non-decreasing and $k_{1}\left(\theta_{i}\right)<1$) for i=1,2 then on applying by Proposition 1(iii) $X_{1}\leq_{rh}X_{2}$ implies $X_{1w_{1}}\leq_{rh}X_{2w_{2}}$.*

**Example** **3** (Additive-Multiplicative weighted distribution)**.** *Let*

$$w\left(x,\theta_{i}\right)=w_{1}\left(x\right)+k_{1}\left(\theta_{i}\right)w_{2}\left(x\right),i=1,2,$$

*where $\theta_{1}\leq \theta_{2}\in \chi$. Suppose that $w_{1}/w_{2}$ is non-increasing (resp. non-decreasing) and $k_{1}$ is non-decreasing (resp. non-increasing). Proposition 1(i) provides that $X_{1}\leq_{lr}X_{2}$ implies $X_{1w_{1}}\leq_{lr}X_{2w_{2}}.$ If, moreover, we assume that $k_{1}\left(\theta_{i}\right)>0,i=1,2$ then when $w_{1}$ and $w_{2}$ are both non-decreasing by Proposition 1(ii) we deduce that $X_{1}\leq_{hr}X_{2}$ gives $X_{1w_{1}}\leq_{hr}X_{2w_{2}}$ and once $w_{1}$ and $w_{2}$ are both non-increasing by Proposition 1(iii) $X_{1}\leq_{rh}X_{2}$ entails that $X_{1w_{1}}\leq_{rh}X_{2w_{2}}$. In a suchlike manner, whenever $k_{1}\left(\theta_{i}\right)\leq 0,$ i = 1, 2 then if $w_{1}$ is non-decreasing and $w_{2}$ is non-increasing, on applying Proposition 1(ii) $X_{1}\leq_{hr}X_{2}$ assures that $X_{1w_{1}}\leq_{hr}X_{2w_{2}}$ and similarly when $w_{1}$ is non-increasing and $w_{2}$ is non-decreasing by Proposition 1(iii) it is deducible that $X_{1}\leq_{rh}X_{2}$ gives $X_{1w_{1}}\leq_{rh}X_{2w_{2}}$.*

**Example** **4** (Weighted left-truncated distribution)**.** *Let*

$$w\left(x,\theta_{i}\right)=w_{1}\left(x\right)I\left[w_{2}\left(x\right)>k_{1}\left(\theta_{i}\right)\right],i=1,2$$

*with $\theta_{1}\leq \theta_{2}\in \chi$. Let us suppose that $w_{2}$ and $k_{1}$ are both non-decreasing (or non-increasing) functions. By Proposition 1(i), $X_{1}\leq_{lr}X_{2}$ implies $X_{1w_{1}}\leq_{lr}X_{2w_{2}}.$ If $w_{1}$, $w_{2}$ and $k_{1}$ are all non-decreasing functions then Proposition 1(ii) establishes that $X_{1}\leq_{hr}X_{2}$ implicate $X_{1w_{1}}\leq_{hr}X_{2w_{2}}$. In a similar manner, if $w_{1}$, $w_{2}$ and $k_{1}$ are all non-increasing functions then from Proposition 1(iii) it is deduced that $X_{1}\leq_{rh}X_{2}$ implies $X_{1w_{1}}\leq_{rh}X_{2w_{2}}$.*

**Example** **5** (Weighted right-truncated distribution)**.** *Let*

$$w\left(x,\theta_{i}\right)=w_{1}\left(x\right)I\left[w_{2}\left(x\right)\leq k_{1}\left(\theta_{i}\right)\right],i=1,2,$$

*in which $\theta_{1}\leq \theta_{2}\in \chi$. We assume that $w_{2}$ and $k_{1}$ are both non-decreasing (or non-increasing) functions. Proposition 1(i) guarantees that $X_{1}\leq_{lr}X_{2}$ implies $X_{1w_{1}}\leq_{lr}X_{2w_{2}}.$ If $w_{1}$ is non-decreasing and $w_{2}$ and $k_{1}$ are non-increasing functions then by Proposition 1(ii) $X_{1}\leq_{hr}X_{2}$ yields $X_{1w_{1}}\leq_{hr}X_{2w_{2}}$. In the dual case, if $w_{1}$ is non-increasing and further $w_{2}$ and $k_{1}$ are both non-decreasing functions then Proposition 1(iii) concludes that $X_{1}\leq_{rh}X_{2}$ gives $X_{1w_{1}}\leq_{rh}X_{2w_{2}}$.*

Some relative stochastic orders including the relative (reversed) hazard rate and relative mean residual life orders have attracted the attention of researchers in the last decade (cf. Di-Crescenzo and Longobardi [20], Kayid et al. [21], Misra and Francis [22], Misra et al. [23], Ding et al. [24], Ding and Zhang [25], Misra and Francis [26] and Misra and Francis [27]). We reminisce about the definition of these orders from Rezaei et al. [28] and Kayid et al. [21] [see, for example, Definition 1(v) and (vi)]. In the next theorem, the study of preservation of the relative hazard rate and the relative reversed hazard rate orders are initiated for a well-known class of semiparamtric distributions. For i=1,2, denote by $h_{iw_{i}}\left(t,\theta_{i}\right)$ (${\tilde{h}}_{iw_{i}}\left(t,\theta_{i}\right)$) the hazard rate (resp. the reversed hazard rate) of $X_{iw_{i}}$, where $w_{i}$ and is supposed to be valid as a weight function. Before stating the result, we introduce some notations. Let $w_{i}\left(t,\theta_{i}\right)=v\left({\bar{F}}_{i}\left(t\right),\theta_{i}\right),i=1,2$ be two appropriate weight functions and set

$$\xi \left(x,\theta \right)=\frac{\int_{0}^{x}v\left(u,\theta \right)\;du}{xv(x,\theta )},\;\;x\in \left[0,1\right],\theta \in \chi .$$

Denote by $\xi^{\prime }\left(x,\theta \right)$ partial derivative of ξ(x,θ) with respect to *x*, that is, $\xi^{\prime }\left(x,\theta \right)=\frac{\partial }{\partial x}\xi \left(x,\theta \right).$ The symbol $\overset{sign}{=}$ is used to denote the similar sign.

**Theorem** **1.** *Suppose that $w_{i}\left(t,\theta_{i}\right)=v\left({\bar{F}}_{i}\left(t\right),\theta_{i}\right),i=1,2$ so that $\theta_{1}\leq \theta_{2}$ and $X_{1}\leq_{hr}X_{2}.$ Let $x\xi^{\prime }\left(x,\theta \right)/\xi \left(x,\theta \right)$ is non-increasing (resp. non-decreasing) in x, for all θ and non-increasing (resp. non-decreasing) in θ, for all x where ξ(x,θ) is non-decreasing (resp. non-increasing) in x, for all θ∈χ. Then, $X_{1}\leq_{rhr}X_{2}$ implies that $X_{1w_{1}}\leq_{rhr}X_{2w_{2}}.$*

**Proof.**  From (2), one has $h_{iw_{i}}\left(i,\theta_{i}\right)=w_{i}\left(t,\theta_{i}\right)h_{i}\left(t\right)/B_{i}\left(t,\theta_{i}\right),$ where

$$\begin{array}{cc} B_{i}\left(t,\theta_{i}\right) & =E[v\left({\bar{F}}_{i}\left(X_{i}\right),\theta_{i}\right)|X_{i}>t]\\ \; & =\frac{1}{{\bar{F}}_{i}\left(t\right)}\int_{t}^{\infty }v\left({\bar{F}}_{i}\left(x\right),\theta_{i}\right)\;dF_{i}\left(x\right)\\ \; & =\frac{1}{{\bar{F}}_{i}\left(t\right)}\int_{0}^{{\bar{F}}_{i}\left(t\right)}v\left(u,\theta_{i}\right)\;du,\;\;i=1,2. \end{array}$$

Thus, for all t>0, we have:

$$\begin{array}{cc} \frac{h_{2w_{2}}\left(t,\theta_{2}\right)}{h_{1w_{1}}\left(t,\theta_{1}\right)} & =\frac{h_{2}\left(t\right)}{h_{1}\left(t\right)}\frac{w_{2}\left(t,\theta_{2}\right)}{w_{1}\left(t,\theta_{1}\right)}\frac{B_{1}\left(t,\theta_{1}\right)}{B_{2}\left(t,\theta_{2}\right)}\\ \; & =\frac{h_{2}\left(t\right)}{h_{1}\left(t\right)}\frac{\int_{0}^{{\bar{F}}_{1}\left(t\right)}v\left(u,\theta_{1}\right)\;du/\left({\bar{F}}_{1}\left(t\right)v\left({\bar{F}}_{1}\left(t\right),\theta_{1}\right)\right)}{\int_{0}^{{\bar{F}}_{2}\left(t\right)}v\left(u,\theta_{2}\right)\;du/\left({\bar{F}}_{2}\left(t\right)v\left({\bar{F}}_{2}\left(t\right),\theta_{2}\right)\right)}.\\ \; & =\frac{h_{2}\left(t\right)}{h_{1}\left(t\right)}\frac{\xi ({\bar{F}}_{1}\left(t\right),\theta_{1})}{\xi ({\bar{F}}_{2}\left(t\right),\theta_{2})}. \end{array}$$

By assumption, $h_{2}\left(t\right)/h_{1}\left(t\right)$ is non-increasing in t>0. It suffices only to prove that:

$$\xi \left({\bar{F}}_{1}\left(t\right),\theta_{1}\right)/\xi \left({\bar{F}}_{2}\left(t\right),\theta_{2}\right)\mathrm{is}\text{non-increasing}\mathrm{in}t>0.$$

The assumption $X_{1}\leq_{hr}X_{2}$ yields $h_{1}\left(t\right)\geq h_{2}\left(t\right),$ for all t>0, which further concludes that ${\bar{F}}_{1}\left(t\right)\leq {\bar{F}}_{2}\left(t\right),$ for all t>0. Therefore,

$$\begin{array}{cc} \frac{d}{dt}\frac{\xi ({\bar{F}}_{1}\left(t\right),\theta_{1})}{\xi ({\bar{F}}_{2}\left(t\right),\theta_{2})} & \overset{sign}{=}f_{2}\left(t\right)\xi^{\prime }\left({\bar{F}}_{2}\left(t\right),\theta_{2}\right)\xi \left({\bar{F}}_{1}\left(t\right),\theta_{1}\right)-f_{1}\left(t\right)\xi^{\prime }\left({\bar{F}}_{1}\left(t\right),\theta_{1}\right)\xi \left({\bar{F}}_{2}\left(t\right),\theta_{2}\right)\\ \; & =\frac{f_{2}\left(t\right)}{{\bar{F}}_{2}\left(t\right)}{\bar{F}}_{2}\left(t\right)\xi^{\prime }\left({\bar{F}}_{2}\left(t\right),\theta_{2}\right)\xi \left({\bar{F}}_{1}\left(t\right),\theta_{1}\right)-\frac{f_{1}\left(t\right)}{{\bar{F}}_{1}\left(t\right)}{\bar{F}}_{1}\left(t\right)\xi^{\prime }\left({\bar{F}}_{1}\left(t\right),\theta_{1}\right)\xi \left({\bar{F}}_{2}\left(t\right),\theta_{2}\right)\\ \; & =h_{2}\left(t\right){\bar{F}}_{2}\left(t\right)\xi^{\prime }\left({\bar{F}}_{2}\left(t\right),\theta_{2}\right)\xi \left({\bar{F}}_{1}\left(t\right),\theta_{1}\right)-h_{1}\left(t\right){\bar{F}}_{1}\left(t\right)\xi^{\prime }\left({\bar{F}}_{1}\left(t\right),\theta_{1}\right)\xi \left({\bar{F}}_{2}\left(t\right),\theta_{2}\right), \end{array}$$

is non-positive (resp. non-negative) for all t>0, if, and only if, for all $x_{1}\leq x_{2}$ and for all $\theta_{1}\leq \theta_{2}$ it holds that

$$\frac{x_{1}\xi^{\prime }\left(x_{1},\theta_{1}\right)}{\xi (x_{1},\theta_{1})}\geq \left(resp.\leq \right)\frac{x_{2}\xi^{\prime }\left(x_{2},\theta_{2}\right)}{\xi (x_{2},\theta_{2})},$$

which is validated by assumption. □

To present the result about the preservation of the relative reversed hazard rate order we introduce some other notation. Let us define for x∈[0,1],

$$\xi^{*}\left(x,\theta \right)=\frac{\int_{x}^{1}v\left(u,\theta \right)\;du}{\left(1-x)v(x,\theta \right)}\;,\theta \in \chi .$$

Suppose $\xi^{*\prime }\left(x,\theta \right)$ stands for the partial derivative of $\xi^{*}\left(x,\theta \right)$ with respect to *x*, that is $\xi^{*\prime }\left(x,\theta \right)=\frac{\partial }{\partial x}\xi^{*}\left(x,\theta \right).$

**Theorem** **2.** Let $w_{i}\left(t,\theta_{i}\right)=v\left({\bar{F}}_{i}\left(t\right),\theta_{i}\right),i=1,2$ so that $\theta_{1}\leq \theta_{2}$ and $X_{1}\leq_{rh}X_{2}.$ If $\left(1-x\right)\xi^{*\prime }\left(x,\theta \right)/\xi^{*}\left(x,\theta \right)$ is non-decreasing (resp. non-increasing) in x, for all θ and non-decreasing (resp. non-increasing) in θ, for all x, in which $\xi^{*}\left(x,\theta \right)$ is non-decreasing (non-increasing) in x for all θ∈χ, then $X_{1}\leq_{rrh}X_{2}$ implies that $X_{1w_{1}}\leq_{rrh}X_{2w_{2}}.$

**Proof.**  In spirit of (3), we can write ${\tilde{h}}_{iw_{i}}\left(i,\theta_{i}\right)=w_{i}\left(t,\theta_{i}\right){\tilde{h}}_{i}\left(t\right)/A_{i}\left(t,\theta_{i}\right),$ in which

$$\begin{array}{cc} A_{i}\left(t,\theta_{i}\right) & =E[v\left({\bar{F}}_{i}\left(X_{i}\right),\theta_{i}\right)|X_{i}\leq t]\\ \; & =\frac{1}{1-{\bar{F}}_{i}\left(t\right)}\int_{0}^{t}v\left({\bar{F}}_{i}\left(x\right),\theta_{i}\right)\;dF_{i}\left(x\right)\\ \; & =\frac{1}{1-{\bar{F}}_{i}\left(t\right)}\int_{{\bar{F}}_{i}\left(t\right)}^{1}v\left(u,\theta_{i}\right)\;du,\;\mathrm{for}\;\mathrm{any}\;i=1,2. \end{array}$$

For all t>0, one obtains

$$\begin{array}{cc} \frac{{\tilde{h}}_{2w_{2}}\left(t,\theta_{2}\right)}{{\tilde{h}}_{1w_{1}}\left(t,\theta_{1}\right)} & =\frac{{\tilde{h}}_{2}\left(t\right)}{{\tilde{h}}_{1}\left(t\right)}\frac{w_{2}\left(t,\theta_{2}\right)}{w_{1}\left(t,\theta_{1}\right)}\frac{A_{1}\left(t,\theta_{1}\right)}{A_{2}\left(t,\theta_{2}\right)}\\ \; & =\frac{{\tilde{h}}_{2}\left(t\right)}{{\tilde{h}}_{1}\left(t\right)}\frac{\int_{{\bar{F}}_{1}\left(t\right)}^{1}v\left(u,\theta_{1}\right)\;du/\left(\left(1-{\bar{F}}_{1}\left(t\right)\right)v\left({\bar{F}}_{1}\left(t\right),\theta_{1}\right)\right)}{\int_{{\bar{F}}_{2}\left(t\right)}^{1}v\left(u,\theta_{2}\right)\;du/\left(\left(1-{\bar{F}}_{2}\left(t\right)\right)v\left({\bar{F}}_{2}\left(t\right),\theta_{2}\right)\right)}.\\ \; & =\frac{{\tilde{h}}_{2}\left(t\right)}{{\tilde{h}}_{1}\left(t\right)}\frac{\xi^{*}\left({\bar{F}}_{1}\left(t\right),\theta_{1}\right)}{\xi^{*}\left({\bar{F}}_{2}\left(t\right),\theta_{2}\right)}. \end{array}$$

Since $X_{1}\leq_{rrh}X_{2}$, thus ${\tilde{h}}_{2}\left(t\right)/{\tilde{h}}_{1}\left(t\right)$ is non-decreasing in t>0. It remains to demonstrate that $\xi^{*}\left({\bar{F}}_{2}\left(t\right),\theta_{1}\right)/\xi^{*}\left({\bar{F}}_{1}\left(t\right),\theta_{2}\right)$ is non-increasing in t>0. It is known that $X_{1}\leq_{hr}X_{2}$ implies ${\tilde{h}}_{1}\left(t\right)\leq {\tilde{h}}_{2}\left(t\right),$ for all t>0, which in turn yields ${\bar{F}}_{1}\left(t\right)\leq {\bar{F}}_{2}\left(t\right),$ for all t>0. For that reason,

$$\begin{array}{cc} \frac{d}{dt}\frac{\xi^{*}\left({\bar{F}}_{2}\left(t\right),\theta_{2}\right)}{\xi^{*}\left({\bar{F}}_{1}\left(t\right),\theta_{1}\right)} & \overset{sign}{=}-f_{2}\left(t\right)\xi^{*\prime }\left({\bar{F}}_{2}\left(t\right),\theta_{2}\right)\xi^{*}\left({\bar{F}}_{1}\left(t\right),\theta_{1}\right)+f_{1}\left(t\right)\xi^{*\prime }\left({\bar{F}}_{1}\left(t\right),\theta_{1}\right)\xi^{*}\left({\bar{F}}_{2}\left(t\right),\theta_{2}\right)\\ \; & =\frac{f_{1}\left(t\right)}{F_{1}\left(t\right)}F_{1}\left(t\right)\xi^{*\prime }\left({\bar{F}}_{1}\left(t\right),\theta_{1}\right)\xi^{*}\left({\bar{F}}_{2}\left(t\right),\theta_{2}\right)-\frac{f_{2}\left(t\right)}{F_{2}\left(t\right)}F_{2}\left(t\right)\xi^{*\prime }\left({\bar{F}}_{2}\left(t\right),\theta_{2}\right)\xi^{*}\left({\bar{F}}_{1}\left(t\right),\theta_{1}\right)\\ \; & ={\tilde{h}}_{1}\left(t\right)F_{1}\left(t\right)\xi^{*\prime }\left({\bar{F}}_{1}\left(t\right),\theta_{1}\right)\xi^{*}\left({\bar{F}}_{2}\left(t\right),\theta_{2}\right)-{\tilde{h}}_{2}\left(t\right)F_{2}\left(t\right)\xi^{*\prime }\left({\bar{F}}_{2}\left(t\right),\theta_{2}\right)\xi^{*}\left({\bar{F}}_{1}\left(t\right),\theta_{1}\right), \end{array}$$

is non-positive (resp. non-negative) for all t>0, if, and only if, for all $x_{1}\leq x_{2}$ and for all $\theta_{1}\leq \theta_{2}$:

$$\frac{\left(1-x_{1}\right)\xi^{*\prime }\left(x_{1},\theta_{1}\right)}{\xi^{*}\left(x_{1},\theta_{1}\right)}\leq \left(\mathrm{resp}.\geq \right)\frac{\left(1-x_{2}\right)\xi^{*\prime }\left(x_{2},\theta_{2}\right)}{\xi^{*}\left(x_{2},\theta_{2}\right)},$$

which holds by assumption. □

The weight functions considered in Theorems 1 and 2 encompass some particular cases which may be of independent interest. In that regard, the following corollary is resulted.

**Corollary** **1.** *Let $w_{i}\left(t\right)=v\left({\bar{F}}_{i}\left(t\right)\right),i=1,2$ such that $X_{1}\leq_{hr}X_{2}$. Set*

$$\xi \left(x\right)=\frac{1}{xv(x)}\int_{0}^{x}v\left(u\right)du\;\;and\;\;\xi^{*}\left(x\right)=\frac{1}{\left(1-x)v(x\right)}\int_{x}^{1}v\left(u\right)du.$$

*(i)*  *If $x\xi^{\prime }\left(x\right)/\xi \left(x\right)$ is non-increasing (resp. non-decreasing) in x∈[0,1], in which ξ(x) is non-decreasing (non-increasing) in x, then $X_{1}\leq_{rhr}X_{2}$ implies that $X_{1w_{1}}\leq_{rhr}X_{2w_{2}}.$*
*(ii)*  If $\left(1-x\right)\xi^{*\prime }\left(x\right)/\xi \left(x\right)$ is non-decreasing (resp. non-increasing) in x∈[0,1], in which $\xi^{*}\left(x\right)$ is non-decreasing (non-increasing) in x, then $X_{1}\leq_{rrh}X_{2}$ implies that $X_{1w_{1}}\leq_{rrh}X_{2w_{2}}.$

**Theorem** **3.** Let $w_{i}\left(t,\theta_{i}\right)=v_{i}\left(\bar{F}\left(t\right),\theta_{i}\right)$ and set $\xi_{i}\left(x,\theta_{i}\right)=\left(1/v_{i}\left(x,\theta_{i}\right)\right)\int_{0}^{x}v_{i}\left(u,\theta_{i}\right)\;du$ and $\xi_{i}^{*}\left(x,\theta_{i}\right)=\left(1/v_{i}\left(x,\theta_{i}\right)\right)\int_{x}^{1}v_{i}\left(u,\theta_{i}\right)\;du$ for i=1,2. Then

*(i)*  *$X_{1w_{1}}\leq_{rhr}X_{2w_{2}}$ if, and only if, $\frac{\xi_{2}\left(x,\theta_{2}\right)}{\xi_{1}\left(x,\theta_{1}\right)}$ is non-increasing in x.*
*(ii)*  $X_{1w_{1}}\leq_{rrh}X_{2w_{2}}$ if, and only if, $\frac{\xi_{2}^{*}\left(x,\theta_{2}\right)}{\xi_{1}^{*}\left(x,\theta_{1}\right)}$ is non-decreasing in x.

**Proof.**  We only prove the assertion (i) as the proof of (ii) is similarly accomplished. Note that analogously as in the proof of Theorem 1, we can get

$$\begin{array}{cc} \frac{h_{2w_{2}}\left(t,\theta_{2}\right)}{h_{1w_{1}}\left(t,\theta_{1}\right)} & =\frac{w_{2}\left(t,\theta_{2}\right)}{w_{1}\left(t,\theta_{1}\right)}\frac{\int_{t}^{\infty }v_{1}\left(\bar{F}\left(x\right),\theta_{1}\right)f\left(x\right)\;dx}{\int_{t}^{\infty }v_{2}\left(\bar{F}\left(x\right),\theta_{2}\right)f\left(x\right)\;dx}\\ \; & =\frac{v_{2}\left(\bar{F}\left(t\right),\theta_{2}\right)}{v_{1}\left(\bar{F}\left(t\right),\theta_{1}\right)}\frac{\int_{0}^{\bar{F}\left(t\right)}v_{1}\left(u,\theta_{1}\right)\;du}{\int_{0}^{\bar{F}\left(t\right)}v_{2}\left(u,\theta_{2}\right)\;du}\\ \; & =\frac{\xi_{1}\left(\bar{F}\left(t\right),\theta_{1}\right)}{\xi_{2}\left(\bar{F}\left(t\right),\theta_{2}\right)},\;\;\mathrm{for}\;\mathrm{all}\;t>0. \end{array}$$

It can be seen that, for all t≥0,

$$\frac{d}{dt}\frac{\xi_{1}\left(\bar{F}\left(t\right),\theta_{1}\right)}{\xi_{2}\left(\bar{F}\left(t\right),\theta_{2}\right)}\overset{sign}{=}f\left(t\right)\left[\xi_{2}^{\prime }\left(\bar{F}\left(t\right),\theta_{2}\right)\xi_{1}\left(\bar{F}\left(t\right),\theta_{1}\right)-\xi_{1}^{\prime }\left(\bar{F}\left(t\right),\theta_{1}\right)\xi_{2}\left(\bar{F}\left(t\right),\theta_{2}\right)\right]$$

which is non-positive if, and only if,

$$\frac{\xi_{2}^{\prime }\left(x,\theta_{2}\right)}{\xi_{2}\left(x,\theta_{2}\right)}\leq \frac{\xi_{1}^{\prime }\left(x,\theta_{1}\right)}{\xi_{1}\left(x,\theta_{1}\right)},\;\;\mathrm{for}\;\mathrm{all}x\in \left[0,1\right],$$

or equivalently if $\xi_{2}\left(x,\theta_{2}\right)/\xi_{1}\left(x,\theta_{1}\right)$ is non-increasing in x∈[0,1] according which the ratio $h_{2w_{2}}\left(t,\theta_{2}\right)/h_{1w_{1}}\left(t,\theta_{2}\right)$ is also non-increasing in t>0, that is, $X_{1w_{1}}\leq_{rhr}X_{2w_{2}}.$ The proof is complete. □

The following corollary is a useful observation in the context of Theorem 3 as it illustrates that a typical parametric family of weighted distributions enjoys the relative hazard rate and the relative reversed hazard rate ordering properties in some cases.

**Corollary** **2.** *Suppose that the random variable $X(\theta_{1})$ and $X(\theta_{2})$ for $\theta_{1},\theta_{2}\in \chi$ have density functions*

$$f_{\theta_{1}}\left(t\right)=\frac{v(\bar{F}\left(t\right),\theta_{1})f\left(t\right)}{\int_{0}^{\infty }v\left(\bar{F}\left(t\right),\theta_{1}\right)f\left(t\right)\;dt}\;\;and\;\;f_{\theta_{2}}\left(t\right)=\frac{v(\bar{F}\left(t\right),\theta_{2})f\left(t\right)}{\int_{0}^{\infty }v\left(\bar{F}\left(t\right),\theta_{2}\right)f\left(t\right)\;dt}.$$

For $\xi \left(x,\theta \right)=\left(1/v\left(x,\theta \right)\right)\int_{0}^{x}v\left(u,\theta \right)\;du$ and $\xi^{*}\left(x,\theta \right)=\left(1/v\left(x,\theta \right)\right)\int_{x}^{1}v\left(u,\theta \right)\;du$, we have:

*(i)*  *$X\left(\theta_{1}\right)\leq_{rhr}X\left(\theta_{2}\right),$ for all $\theta_{1}\leq \theta_{2}\in \chi$ if, and only if, ξ(x,θ) is RR${}_{2}$ in (x,θ).*
*(ii)*  $X\left(\theta_{1}\right)\leq_{rrh}X\left(\theta_{2}\right),$ for all $\theta_{1}\leq \theta_{2}\in \chi$ if, and only if, $\xi^{*}\left(x,\theta \right)$ TP${}_{2}$ in (x,θ).

In reliability and survival theories, feature of ordering for lifetime of coherent systems is a relevant subject to be studied. To this end, Navarro et al. [29] obtained a representation of the system reliability ${\bar{F}}_{Sys}$ as a distorted function of the common component reliability $\bar{F}$ such that ${\bar{F}}_{Sys}\left(t\right)=\bar{F}\left(t\right)$, where *h* is an non-decreasing function depending on the structure of the underlying system and the survival copula of the joint distribution of the component lifetimes. In this context, they have shown that the reliability function of a coherent system with dependent identically distributed (DID) components can be written as a distorted function of the common component reliability function. The following lemma is due to Navarro et al. [29].

**Lemma** **1.** *Let τ(X) be the lifetime of a coherent system formed by n DID components with the vector of random lifetimes $X=(X_{1},X_{2},\ldots ,X_{n})$ with common survival function $\bar{F}$. Then the reliability function of τ(X) can be written as*

$${\bar{F}}_{\tau (X)}\left(x\right)=h\left(\bar{F}\left(x\right)\right),$$

*where h(·):[0,1]→[0,1] is an non-decreasing continuous function such that h(0)=0 and h(1)=1. The function h is called the domination (or distortion) function which is characterized through the structure function ϕ(·) of the system (see, e.g., Barlow and Proschan [30]) and on the survival copula $\hat{C}$ of $X_{1},X_{2},\ldots ,X_{n}$.*

In the set up of the particular weighted distributions given in Section 3.2, the survival function of the arisen weighted distribution can be commuted to a distorted survival function, as specified earlier in Lemma 1, for which the domination function is characterized by the associated weight function. To this purpose, consider the weight function $w\left(x,\theta \right)=v(\bar{F}\left(x\right),\theta )$ and notice that in this case $X_{w}$ has the survival function

$${\bar{F}}_{w}\left(t,\theta \right)=\frac{\int_{t}^{\infty }v\left(\bar{F}\left(x\right),\theta \right)\;dF\left(x\right)}{\int_{-\infty }^{\infty }v\left(\bar{F}\left(x\right),\theta \right)\;dF\left(x\right)}=\frac{\int_{0}^{\bar{F}\left(t\right)}v\left(u,\theta \right)\;du}{\int_{0}^{1}v\left(u,\theta \right)\;du}=h_{\theta }\left(\bar{F}\left(t\right)\right),$$

where

$$h_{\theta }\left(x\right)=\frac{\int_{0}^{x}v\left(u,\theta \right)\;du}{\int_{0}^{1}v\left(u,\theta \right)\;du},\;\;x\in \left[0,1\right]$$

plays the role of a parametric domination function. Note that $h_{\theta }\left(\cdot \right):\left[0,1\right]\to \left[0,1\right]$ is a non-decreasing continuous function with $h_{\theta }\left(0\right)=0$ and $h_{\theta }\left(1\right)=1$. In the reversed direction, if $h_{\theta }$ is a distortion (domination) function and $v\left(x,\theta \right)=h_{\theta }^{\prime }\left(x\right),$ for any x∈[0,1] then $\int_{0}^{1}v\left(x,\theta \right)\;dx=1$ and thus ${\bar{F}}_{w}\left(t,\theta \right)=h_{\theta }\left(\bar{F}\left(t\right)\right).$ Therefore, there is a unique relationship between v(·,θ) and $h_{\theta }\left(\cdot \right)$ that is the studies of weighted distributions in the context of semiparametric models entertain the studies of distorted survival functions and vice versa. The parameter θ may be an appropriate quantity that affects the magnitude of system’s lifetime. In the case when DID components construct the system, θ may be related to the dependency of the component lifetimes in a way that the survival copula in Lemma 1 depends on θ. For instance, in the case where the Archimedean copula or the FGM copula is adopted to model the association of lifetime of components in a coherent system. The following results are useful to analysis of relative ordering properties of coherent systems as to the best of our knowledge such a study has not been developed in the literature thus far. The following proposition is a direct consequence of Theorem 3.

**Proposition** **2.** Let $\xi_{i}\left(x\right)=h_{i}\left(x\right)/h_{i}^{\prime }\left(x\right)$ and $\xi_{i}^{*}\left(x\right)=\left(1-h_{i}\left(x\right)\right)/h_{i}^{\prime }\left(x\right)$ for i=1,2 where $h_{1}$ and $h_{2}$ are two domination functions. Let $T_{1}$ and $T_{2}$ have respective survival functions $h_{1}\left(\bar{F}\right)$ and $h_{2}\left(\bar{F}\right).$ Then,

*(i)*  *$T_{1}\leq_{rhr}T_{2},$ if, and only if, $\xi_{i}\left(x\right)$ is RR${}_{2}$ in (i,x)∈{1,2}×[0,1].*
*(ii)*  $T_{1}\leq_{rrh}T_{2},$ if, and only if, $\xi_{i}^{*}\left(x\right)$ is TP${}_{2}$ in (i,x)∈{1,2}×[0,1].

The following example illustrates an application of Proposition 2.

**Example** **6.** *Suppose that $X_{i},\;i=1,2,3,4$ denote the lifetime of four components having survival function $\bar{F}$. Let*

$$T_{1}=\max\left\{\min\left\{X_{1},X_{2}\right\},\min\left\{X_{2},X_{3}\right\},\min\left\{X_{3},X_{4}\right\}\right\}\;\;and\;\;T_{2}=\max\left\{\min\left\{X_{1},X_{2}\right\},\min\left\{X_{3},X_{4}\right\}\right\}$$

*denote the lifetime of two coherent systems. According to Table I in Navarro et al. [29], when $X_{i}$’s are independent, $T_{1}$ and $T_{2}$ have survival functions $h_{1}\left(\bar{F}\right)$ and $h_{2}\left(\bar{F}\right),$ respectively, in which $h_{1}\left(u\right)=3u^{2}-2u^{3}$ and $h_{2}\left(u\right)=2u^{2}-u^{4}$. For all x∈[0,1], we have*

$$\frac{d}{dx}\frac{\xi_{2}\left(x\right)}{\xi_{1}\left(x\right)}=\frac{d}{dx}\frac{2-x^{2}}{\left(1+x)(3-2x\right)}=-\frac{1+{\left(1-x\right)}^{2}}{{\left(\left(1+x\right)\left(3-2x\right)\right)}^{2}}\leq 0.$$

*Hence, $\xi_{i}\left(x\right)$ is RR${}_{2}$ in (i,x)∈{1,2}×[0,1]. That is, Proposition 2(i) concludes that $T_{1}\leq_{rhr}T_{2}$.*

In the following example, we show that Proposition 2 can also be applied to systems with DID components.

**Example** **7.** *Suppose that $\left(X_{1},X_{2},X_{3}\right)$ are dependent with a Farlie-Gumbel-Morgenstern (FGM) joint reliability function given by*

$$\bar{F}\left(x_{1},x_{2},x_{3}\right)=\bar{F}\left(x_{1}\right)\bar{F}\left(x_{2}\right)\bar{F}\left(x_{3}\right)\left(1-\alpha F\left(x_{1}\right)F\left(x_{2}\right)F\left(x_{3}\right)\right),$$

*where α∈[−1,1], F the marginal distribution of $X_{i}$’s and $\bar{F}=1-F$. Consider the parallel system with lifetime $T_{\alpha }=\max\left\{X_{1},X_{2},X_{3}\right\}$ for which*

$$\begin{array}{cc} {\bar{F}}_{\alpha }\left(x\right) & =\bar{F}\left(x,x,x\right)-3\bar{F}\left(x,x,0\right)+3\bar{F}\left(x\right)\\ \; & ={\bar{F}}^{3}\left(x\right)-\alpha {\left(\bar{F}\left(x\right)-{\bar{F}}^{2}\left(x\right)\right)}^{3}-3{\bar{F}}^{2}\left(x\right)+3\bar{F}\left(x\right). \end{array}$$

**Figure 1:**
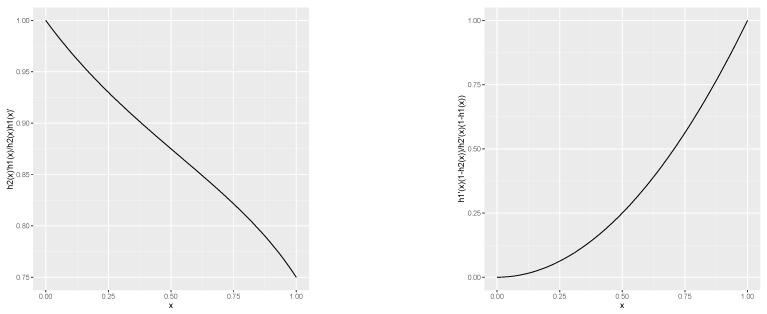
Plot of $\xi_{2}\left(x\right)/\xi_{1}\left(x\right)$ (**left**); Plot of $\xi_{2}^{*}\left(x\right)/\xi_{1}^{*}\left(x\right)$ (**right**).

Thus, ${\bar{F}}_{\alpha }\left(x\right)=h_{\alpha }\left(\bar{F}\left(x\right)\right),$ where $h_{\alpha }\left(u\right)=u^{3}-\alpha {\left(u-u^{2}\right)}^{3}-3u^{2}+3u.$ It can be shown that if $h_{1}\left(x\right)=x^{3}-0.5{\left(x-x^{2}\right)}^{3}-3x^{2}+3x$ and $h_{2}\left(x\right)=x^{3}-0.75{\left(x-x^{2}\right)}^{3}-3x^{2}+3x$ then $\xi_{i}^{*}\left(x\right)=\left(1-h_{i}\left(x\right)\right)/h_{i}^{\prime }\left(x\right)$ is TP${}_{2}$ in (i,x)∈{1,2}×[0,1] because the function $\xi_{2}^{*}\left(x\right)/\xi_{1}^{*}\left(x\right)$ is non-decreasing in x∈[0,1] as the Figure 1 indicates. Hence, according to Proposition 2(ii) one obtains $T_{0.5}\leq_{rrh}T_{0.75}.$

The following example reveals a relative ordering property in the Marshall-Olkin family of distributions.

**Example** **8.** *Suppose that the incorporated weight function is*

$$w\left(x,\theta \right)=v\left(\bar{F}\left(x\right),\theta \right)=\frac{1}{{\left(1-\bar{\theta }\bar{F}\left(x\right)\right)}^{2}},$$

*where $\bar{\theta }=1-\theta$ and θ>0. The random variable $X_{w}$ has survival function*

(8) $${\bar{F}}_{w}\left(x,\theta \right)=\frac{\theta \bar{F}\left(x\right)}{\left(1-\bar{\theta }\bar{F}\left(x\right)\right)}=h_{\theta }\left(\bar{F}\left(x\right)\right),$$

*so that $h_{\theta }\left(u\right)=\theta u/\left(1-\bar{\theta }u\right)$ which is considered to be the relevant domination function. Note that the family of distributions characterized via (8) is called the proportional odds family of distributions which is due to Marshall and Olkin [31]. Let $T_{1}$ and $T_{2}$ be two random variables with respective survival functions $h_{\theta_{1}}\left(\bar{F}\right)$ and $h_{\theta_{2}}\left(\bar{F}\right)$ such that $\theta_{1}>\theta_{2}.$ It can be seen that*

$$\xi_{i}\left(x\right)=\frac{h_{\theta_{i}}\left(x\right)}{h_{\theta_{i}}^{\prime }\left(x\right)}=x\left(1-\bar{\theta_{i}}x\right),\;\;i=1,2.$$

*It follows that*

$$\frac{d}{dx}\frac{\xi_{2}\left(x\right)}{\xi_{1}\left(x\right)}=\frac{\theta_{2}-\theta_{1}}{{\left(1-\bar{\theta_{1}}x\right)}^{2}}<0,$$

*that is $\xi_{i}\left(x\right)$ is RR${}_{2}$ in i=1,2 and x>0. Thus, according to Proposition 2(i) we deduce that $T_{1}\leq_{rhr}T_{2}.$*

### 4.2. Comparisons of Mixture Weighted Distribution

In this segment, the problem of preservation of a number of stochastic orderings in the mixture weighted model is investigated. The study is carried out in two different settings, where firstly the random parameter varies in distribution while the underlying distribution remains unchanged and secondly the underlying distribution is changed in the case when the random parameter is fixed in distribution. The results obtained by Kayid et al. [32] are developed to entertain more dynamic weighted distributions.

It is followed up that some stochastic orders of random parameters as well as the underlying random variables are transmitted to the random variables with the associated mixture weighted distribution. Give thought to $\Theta_{i}$ as a random variable with the pdf $g_{i}$, the cdf $G_{i}$ and the sf ${\bar{G}}_{i}=1-G_{i},$ for i=1,2. Contemplate the random variable $X_{i}^{*},i=1,2$ having pdf

(9) $$f_{i}^{*}\left(x\right)=f\left(x\right)\int_{\theta \in \chi }\frac{w(x,\theta )}{\mu (\theta )}g_{i}\left(\theta \right)\;d\theta =f\left(x\right)\;E\left[\frac{w(x,\Theta_{i})}{\mu (\Theta_{i})}\right]$$

from which the cdf $F_{i}^{*}$ and the sf ${\bar{F}}_{i}^{*}=1-F_{i}^{*}$ of $X_{i}^{*}$ are procured after somewhat plain algebraic calculations, respectively, by

(10) $$F_{i}^{*}\left(x\right)=F\left(x\right)E\left(\frac{A(x,\Theta_{i})}{\mu (\Theta_{i})}\right)\;\;\mathrm{and}\;\;{\bar{F}}_{i}^{*}\left(x\right)=\bar{F}\left(x\right)E\left(\frac{B(x,\Theta_{i})}{\mu (\Theta_{i})}\right),\;i=1,2,$$

where the bivariate functions *A* and *B* and the function μ are all determined as earlier in Section 1. In the rest of the paper, it is taken for granted that the random variables $\Theta_{1}$ and $\Theta_{2}$ are independent. Denote by $h_{i}^{*}$ and ${\tilde{h}}_{i}^{*}$ the hazard rate and the reversed hazard rate of $X_{i}^{*}$, respectively. It can be seen, after some integral calculation, that

(11) $$h_{i}^{*}\left(x\right)=\int_{\theta \in \chi }\frac{f_{w}\left(x,\theta \right)}{{\bar{F}}_{i}^{*}\left(x\right)}\;dG_{i}\left(\theta \right)=h\left(x\right)\;E\left[\left.\frac{w(x,\Theta_{i})}{B(x,\Theta_{i})}\;\right|X_{i}^{*}>x\right]$$

and

(12) $${\tilde{h}}_{i}^{*}\left(x\right)=\int_{\theta \in \chi }\frac{f_{w}\left(x,\theta \right)}{F_{i}^{*}\left(x\right)}\;dG_{i}\left(\theta \right)=\tilde{h}\left(x\right)\;E\left[\left.\frac{w(x,\Theta_{i})}{A(x,\Theta_{i})}\;\right|X_{i}^{*}\leq x\right]$$

The following result demonstrates the likelihood ratio order preservation in the model (9).

**Theorem** **4.** Let w(x,θ) be TP${}_{2}$ (resp. RR${}_{2}$) in (x,θ)∈ζ×χ. Then, $\Theta_{1}\leq_{lr}\Theta_{2}$ implies $X_{1}^{*}\leq_{lr}\left(resp.\geq_{lr}\right)X_{2}^{*}$.

**Proof.**  It is not impenetrable to realize that $X_{1}^{*}\leq_{lr}X_{2}^{*}$ if, and only if, $f_{i}^{*}\left(x\right)$ is TP${}_{2}$ in (i,x)∈{1,2}×ζ. In spirit of (9), one gets

$$f_{i}^{*}\left(x\right)=\int_{\theta \in \chi }f_{w}\left(x,\theta \right)g_{i}\left(\theta \right)\;d\theta =\int_{\theta \in \chi }\frac{w(x,\theta )f(x)}{\mu (\theta )}g_{i}\left(\theta \right)\;d\theta ,i=1,2.$$

By the assumption of $\Theta_{1}\leq_{lr}\Theta_{2}$ we can rely on the fact that $g_{i}\left(\theta \right)$ is TP${}_{2}$ (resp. RR${}_{2}$) in (i,θ)∈{1,2}×χ. It is also obvious that $\frac{f(x)w(x,\theta )}{\mu (\theta )}$ is TP${}_{2}$ in (x,θ)∈ζ×χ. The general composition theorem of Karlin [15] concludes the desired result. □

**Theorem** **5.** Let w(x,θ) be TP${}_{2}$ (resp. RR${}_{2}$) in (x,θ)∈ζ×χ. Then, $\Theta_{1}\leq_{hr}\Theta_{2}$ implies $X_{1}^{*}\leq_{hr}\left(resp.\geq_{hr}\right)X_{2}^{*}$.

**Proof.**  We prove the non-parenthetical part. The parenthetical part of the theorem can be similarly proved. In consideration of the second identity in (10) one observes

$${\bar{F}}_{i}^{*}\left(x\right)=\int_{\theta \in \chi }\Psi \left(\theta ,x\right)\;dG_{i}\left(\theta \right),\;\;i=1,2;$$

where

$$\Psi \left(\theta ,x\right)=\frac{\bar{F}\left(x\right)\;B\left(x,\theta \right)}{\mu (\theta )}.$$

Take into account that

$$\begin{array}{cc} B(x,\theta ) & =\int_{x}^{\infty }w\left(x^{\prime },\theta \right)\frac{f(x^{\prime })}{\bar{F}\left(x\right)}\;dx^{\prime }\\ \; & =\int_{-\infty }^{\infty }\Phi \left(x,x^{\prime }\right)w\left(x^{\prime },\theta \right)\;dx^{\prime } \end{array}$$

such that

$$\Phi \left(x,x^{\prime }\right)=\left\{\begin{array}{cc} 0, & \mathrm{for}\;x^{\prime }\leq \;x\\ \;\\ \frac{f(x^{\prime })}{\bar{F}\left(x\right)}, & \mathrm{for}\;x^{\prime }>x. \end{array}\right.$$

By assumption $w(x^{\prime },\theta )$ is TP${}_{2}$ in $(x^{\prime },\theta )\in \zeta \times \chi$ and $\Phi (x,x^{\prime })$ is TP${}_{2}$ in $(x,x^{\prime })\in \zeta \times \zeta .$ Hence, the general composition theorem of Karlin [15] concludes that B(x,θ) is TP${}_{2}$ in (x,θ)∈ζ×χ. Since μ(θ)=B(−∞,θ) and since B(x,θ) is TP${}_{2}$ in (x,θ), then B(x,θ)/μ(θ) is non-decreasing in θ∈χ thus Ψ(θ,x) is TP${}_{2}$ in (θ,x)∈χ×ζ and further it is non-decreasing in θ∈χ. It can be readily claimed by assumption that ${\bar{G}}_{i}\left(\theta \right)$ is TP${}_{2}$ in (i,θ)∈{1,2}×χ. On account of Lemma 4.2 in Li and Xu [33] we deduce that ${\bar{F}}_{i}^{*}\left(x\right)$ is TP${}_{2}$ in (i,x)∈{1,2}×ζ and the result follows. □

In the setup of the model (9), the reversed hazard rate order of the random parameters is relocated into the overall random variables.

**Theorem** **6.** Let w(x,θ) be TP${}_{2}$ (resp. RR${}_{2}$) in (x,θ)∈ζ×χ. Then, $\Theta_{1}\leq_{rh}\Theta_{2}$ implies $X_{1}^{*}\leq_{rh}\left(resp.\geq_{rh}\right)X_{2}^{*}$.

**Proof.**  The non-parenthetical part is only proved since the proof for the parenthetical part is analogously carried out. In view of the former identity in (10), it is inferred that $X_{1}^{*}\leq_{rh}X_{2}^{*}$ if, and only if,

$$E\left[\frac{A(x_{1},\Theta_{1})}{\mu (\Theta_{1})}\;\frac{A(x_{2},\Theta_{2})}{\mu (\Theta_{2})}\right]\geq E\left[\frac{A(x_{1},\Theta_{2})}{\mu (\Theta_{2})}\;\frac{A(x_{2},\Theta_{1})}{\mu (\Theta_{1})}\right]$$

for all $x_{1}\leq x_{2}\in \zeta .$ Let us explicate that

$$\phi_{1}\left(\theta_{1},\theta_{2}\right)=\frac{A(x_{1},\theta_{2})}{\mu (\theta_{2})}\times \frac{A(x_{2},\theta_{1})}{\mu (\theta_{1})},$$

and

$$\phi_{2}\left(\theta_{1},\theta_{2}\right)=\frac{A(x_{1},\theta_{1})}{\mu (\theta_{1})}\times \frac{A(x_{2},\theta_{2})}{\mu (\theta_{2})}.$$

It can be seen that

$$\begin{array}{cc} A(x,\theta ) & =\int_{0}^{x}w\left(x^{\prime },\theta \right)\frac{f(x^{\prime })}{F(x)}\;dx^{\prime }\\ \; & =\int_{0}^{\infty }\phi \left(x,x^{\prime }\right)w\left(x^{\prime },\theta \right)\;\;dx^{\prime } \end{array}$$

so that

$$\phi \left(x,x^{\prime }\right)=\left\{\begin{array}{cc} \frac{f(x^{\prime })}{F(x)}, & \mathrm{for}\;x^{\prime }\leq \;x\\ \;\\ 0, & \mathrm{for}\;x^{\prime }>x. \end{array}\right.$$

From assumption $w(x^{\prime },\theta )$ is TP${}_{2}$ in $(x^{\prime },\theta )\in \zeta \times \chi$ and $\phi (x,x^{\prime })$ is TP${}_{2}$ in $(x,x^{\prime })\in \zeta \times \zeta .$ The general composition theorem of Karlin [15] therefore is applied to draw the inference that A(x,θ) is TP${}_{2}$ in (x,θ)∈ζ×χ. For that reason, for all $\theta_{1}\leq \theta_{2}\in \chi$ and $x_{1}\leq x_{2}\in \zeta$

$$\begin{array}{cc} \Delta \phi_{21}\left(\theta_{1},\theta_{2}\right) & =\phi_{2}\left(\theta_{1},\theta_{2}\right)-\phi_{1}\left(\theta_{1},\theta_{2}\right)\\ \; & =\frac{1}{\mu \left(\theta_{1}\right)\mu \left(\theta_{2}\right)}\left[A\left(x_{1},\theta_{1}\right)A\left(x_{2},\theta_{2}\right)-A\left(x_{1},\theta_{2}\right)A\left(x_{2},\theta_{1}\right)\right]\\ \; & \geq 0. \end{array}$$

It is clearly seen that $\mu \left(\theta_{i}\right)=A\left(\infty ,\theta_{i}\right),$ for i=1,2. Since A(x,θ) is TP${}_{2}$ in (x,θ)∈ζ×χ thus $A\left(x_{1},\theta_{1}\right)/\mu \left(\theta_{1}\right)$ is non-increasing in $\theta_{1}\in \chi$ and in addition $A\left(x_{2},\theta_{1}\right)/\mu \left(x_{1},\theta_{1}\right)$ is non-decreasing in $\theta_{1}\chi .$ On that account,

$$\Delta \phi_{21}\left(\theta_{1},\theta_{2}\right)=\frac{A(x_{1},\theta_{1})}{\mu \left(\theta_{1}\right)\mu \left(\theta_{2}\right)}\left[A\left(x_{2},\theta_{2}\right)-A\left(x_{1},\theta_{2}\right)\frac{A(x_{2},\theta_{1})}{A(x_{1},\theta_{1})}\right]$$

is non-negative and also non-increasing in $\theta_{1}\in \chi ,$ for all $\theta_{1}\leq \theta_{2}\in \chi$ and for all $x_{1}\leq x_{2}\in \zeta .$ The proof is completed by Theorem 1.B.48 of Shaked and Shanthikumar [34]. □

The weight functions brought in Section 3.1 and Section 3.2 are all TP${}_{2}$ (or RR${}_{2}$) at least under some (mild) condition. That is, they are applicable to develop the $\leq_{lr},$ the $\leq_{hr}$ and the $\leq_{rh}$ orders from the random parameter into the mixture (average) variable in the model of (9) according to the result of Theorems 4–6, respectively. It is remarkable that

(i) $w\left(x,\theta \right)=w_{1}\left(x\right){\left(w_{2}\left(x\right)\right)}^{k_{1}\left(\theta \right)}$ is TP${}_{2}$ in (x,θ)∈ζ×χ whenever $w_{2}\left(x\right)$ is non-decreasing (resp. non-increasing) in x∈ζ and $k_{1}\left(\theta \right)$ is non-decreasing (resp. non-increasing) in θ∈χ.
(ii) $w\left(x,\theta \right)=w_{1}\left(x\right){\left(k_{1}\left(\theta \right)\right)}^{w_{2}\left(x\right)}$ is TP${}_{2}$ in (x,θ)∈ζ×χ whenever $w_{2}\left(x\right)$ is non-decreasing (resp. non-increasing) in x∈ζ and $k_{1}\left(\theta \right)$ is non-decreasing (resp. non-increasing) in θ∈χ.
(iii) $w\left(x,\theta \right)=w_{1}\left(x\right)k_{1}\left(\theta \right)$ is TP${}_{2}$ in (x,θ)∈ζ×χ.
(iv) $w\left(x,\theta \right)=w_{1}\left(x\right)+k_{1}\left(\theta \right)w_{2}\left(x\right)$ is TP${}_{2}$ in (x,θ)∈ζ×χ whenever $w_{1}\left(x\right)/w_{2}\left(x\right)$ is non-increasing (resp. non-decreasing) in x∈ζ and $k_{1}\left(\theta \right)$ is non-decreasing (resp. non-increasing) in θ∈χ.
(v) $w\left(x,\theta \right)=w_{1}\left(x\right)I\left[w_{2}\left(x\right)>k_{1}\left(\theta \right)\right]$ is TP${}_{2}$ in (x,θ)∈ζ×χ whenever $w_{2}\left(x\right)$ is non-decreasing (resp. non-increasing) in x∈ζ and $k_{1}\left(\theta \right)$ is non-decreasing (resp. non-increasing) in θ∈χ.
(vi) ($w\left(x,\theta \right)=w_{1}\left(x\right)I\left[w_{2}\left(x\right)\leq k_{1}\left(\theta \right)\right]$ is TP${}_{2}$ in (x,θ)∈ζ×χ whenever $w_{2}\left(x\right)$ is non-decreasing (resp. non-increasing) in x∈ζ and $k_{1}\left(\theta \right)$ is non-decreasing (resp. non-increasing) in θ∈χ.
(vii) $w\left(x,\theta \right)={\bar{F}}^{\theta -1}\left(x\right)$ is RR${}_{2}$ in $\left(x,\theta \right)\in \zeta \times R^{+}.$
(viii) $w\left(x,\theta \right)=F^{\theta -1}\left(x\right)$ is TP${}_{2}$ in $\left(x,\theta \right)\in \zeta \times R^{+}$.
(ix) $w\left(x,\theta \right)=1/({\left(1-\bar{\theta }\bar{F}\left(x\right)\right)}^{2})$ is TP${}_{2}$ in $\left(x,\theta \right)\in \zeta \times R^{+}$.
(x) $w\left(x,\theta \right)=-\ln^{\theta }\left(\bar{F}\left(x\right)\right)$ is TP${}_{2}$ in (x,θ)∈ζ×N.
(xi) $w\left(x,\theta \right)=-\ln^{\theta }\left(F\left(x\right)\right)$ is RR${}_{2}$ in (x,θ)∈ζ×N.
(xii) w(x,θ)=f(θ+x)/f(x) is TP${}_{2}$ in $\left(x,\theta \right)\in \zeta \times R^{+}$ whenever *f*, as the density function of *X*, is log-convex on ζ and it is RR${}_{2}$ in $\left(x,\theta \right)\in \zeta \times R^{+}$ provided that *f* is log-concave on ζ.
(xiii) w(x,θ)=f(θ−x)/f(x) is RR${}_{2}$ in $\left(x,\theta \right)\in \zeta \times R^{+}$ whenever *f* is log-convex on ζ and it is TP${}_{2}$ in $\left(x,\theta \right)\in \zeta \times R^{+}$ when *f* is log-concave on ζ.
(xiv) w(x,θ)=f(θx)/f(x) is TP${}_{2}$ in $\left(x,\theta \right)\in \zeta \times R^{+}$ whenever ln(X) has a log-convex density function while it is RR${}_{2}$ in $\left(x,\theta \right)\in \zeta \times R^{+}$ provided that ln(X) has a log-concave density function.

In a modified setup of the mixture weighted model, we consider the case where $X_{i}$ is a lifespan with pdf $f_{i}$ and cdf $F_{i},$ for i=1,2. Then $X_{i}^{**}$ is taken as the random variable with average density

(13) $$f_{i}^{**}\left(x\right)=f_{i}\left(x\right)\int_{\theta \in \chi }\frac{w(x,\theta )}{\mu_{i}\left(\theta \right)}g\left(\theta \right)\;d\theta =f_{i}\left(x\right)\;E\left[\frac{w(x,\Theta )}{\mu_{i}\left(\Theta \right)}\right],$$

where *g* stands for the pdf of Θ and $\mu_{i}\left(\theta \right)=E\left[w\left(X_{i},\theta \right)\right],$ for i=1,2. In the new setting with the mixture model given in (13) two baseline variables $X_{1}$ and $X_{2}$ are implicated. The random lifetimes $X_{1}^{**}$ and $X_{2}^{**}$ are assumed to have density functions $f_{1}^{**}$ and $f_{1}^{**},$ respectively. The mixing variable Θ shares an equal impact upon the construction of the mixture densities.

**Theorem** **7.** Let (Θ|$X_{1}^{**}>x)\leq_{st}(\Theta$|$X_{2}^{**}>x),$ for all x>0. Let w(x,θ) be TP${}_{2}$ in (x,θ)∈ζ×χ and that it is non-decreasing in x∈ζ, for all θ∈χ. Then $X_{1}\leq_{hr}X_{2}$ implies $X_{1}^{**}\leq_{hr}X_{2}^{**}$.

**Proof.**  For i=1,2, suppose that $h_{i}$ and $h_{i}^{**}$ represent the hazard rate functions of $X_{i}$ and $X_{i}^{**},$ respectively. In a same manner as in (12),

$$h_{i}^{**}\left(x\right)=h_{i}\left(x\right)\;E\left[\frac{w(x,\Theta )}{B_{i}\left(x,\Theta \right)}\;|\;X_{i}^{**}>x\right],$$

where $B_{i}\left(x,\theta \right)=E(w\left(X_{i},\theta \right)$|$X_{i}>x),$ for i=1,2. It is known that $X_{1}\leq_{hr}X_{2}$ if, and only if, $(X_{1}$|$X_{1}>x)\leq_{st}(X_{2}$|$X_{2}>x),$ for all x>0. Therefore,

$$\begin{array}{lll} B_{1}\left(x,\theta \right) & = & E(w\left(X_{1},\theta \right)\;|\;X_{1}>x)\\ & \leq & B_{2}\left(x,\theta \right)=E\left(w\left(X_{2},\theta \right)\;|\;X_{2}>x\right), \end{array}$$

for all x>0 and θ∈χ. By assumption, $h_{1}\left(x\right)\geq h_{2}\left(x\right),$ for all x>0. Hence,

(14) $$\begin{array}{cc} h_{1}^{**}\left(x\right)-h_{2}^{**}\left(x\right) & =\int_{0}^{\infty }\frac{w\left(x,\theta \right)h_{1}\left(x\right)}{B_{1}\left(x,\theta \right)}\;d\Pi \left(\theta \;|\;X_{1}^{**}>x\right)\\ \; & -\int_{0}^{\infty }\frac{w\left(x,\theta \right)h_{2}\left(x\right)}{B_{2}\left(x,\theta \right)}\;d\Pi \left(\theta \;|\;X_{2}^{**}>x\right)\\ \; & \;\;\\ \; & \geq h_{2}\left(x\right)\;\int_{0}^{\infty }\frac{w(x,\theta )}{B_{2}\left(x,\theta \right)}\;d\left[\Pi \left(\theta \;|\;X_{1}^{**}>x\right)-\Pi \left(\theta \;|\;X_{2}^{**}>x\right)\right]. \end{array}$$

Since $w\left(x,\theta \right)/B_{2}\left(x,\theta \right)$ is non-increasing in θ∈χ, thus by assumption,

$$\int_{0}^{T}d\left[\Pi \left(\theta \;|\;X_{1}^{**}>x\right)-\Pi \left(\theta \;|\;X_{2}^{**}>x\right)\right]\geq 0,$$

for all T>0. By Lemma 7.1(b) of Barlow and Proschan [30] to (14) we attain the proof. □

The last result establishes the reversed hazard rate ordering preservation in the baseline-varied mixture weighted model of (13).

**Theorem** **8.** Let (Θ|$X_{1}^{**}\leq x)\leq_{st}(\Theta$|$X_{2}^{**}\leq x),$ for all x>0, and let $(X_{1}^{**}$|$\Theta =\theta_{1})\leq_{rh}(X_{2}^{**}$|$\Theta =\theta_{2}),$ for all $\theta_{1}\leq \theta_{2}\in \chi .$ Furthermore, let w(x,θ) be TP${}_{2}$ in (x,θ)∈ζ×χ. Then $X_{1}\leq_{rh}X_{2}$ implies $X_{1}^{**}\leq_{rh}X_{2}^{**}$.

**Proof.**  First, we denote by ${\tilde{h}}_{i}$ and ${\tilde{h}}_{i}^{**}$ the reversed hazard rate functions of $X_{i}$ and $X_{i}^{**},$ respectively, for i=1,2. For all x>0,

$${\tilde{h}}_{i}^{**}\left(x\right)={\tilde{h}}_{i}\left(x\right)\;E\left[\frac{w(x,\Theta )}{A_{i}\left(x,\Theta \right)}\;|\;X_{i}^{**}\leq x\right],$$

where $A_{i}\left(x,\theta \right)=E\left[w\left(X_{i},\theta \right)\;|\;X_{i}\leq x\right],$ for i=1,2. The order relation $(X_{1}^{**}$|$\Theta =\theta_{1})\leq_{rh}(X_{2}^{**}$|$\Theta =\theta_{2}),$ for all $\theta_{1}\leq \theta_{2}\in \chi$ yields

$$\begin{array}{cc} \frac{w\left(x,\theta \right){\tilde{h}}_{2}\left(x\right)}{A_{2}\left(x,\theta \right)} & =\frac{w\left(x,\theta \right)f_{2}\left(x\right)}{\int_{0}^{x}w\left(x^{\prime },\theta \right)f_{2}\left(x^{\prime }\right)\;dx^{\prime }}\\ \; & \geq \frac{w\left(x,\theta \right)f_{1}\left(x\right)}{\int_{0}^{x}w\left(x^{\prime },\theta \right)f_{1}\left(x^{\prime }\right)\;dx^{\prime }}\\ \; & =\frac{w\left(x,\theta \right){\tilde{h}}_{1}\left(x\right)}{A_{1}\left(x,\theta \right)}, \end{array}$$

for all x>0, and θ∈χ. Thus

(15) $$\begin{array}{cc} {\tilde{h}}_{2}^{**}\left(x\right)-{\tilde{h}}_{1}^{**}\left(x\right) & =\int_{0}^{\infty }\frac{w\left(x,\theta \right){\tilde{h}}_{2}\left(x\right)}{A_{2}\left(x,\theta \right)}\;d\Pi \left(\theta \;|\;X_{2}^{**}\leq x\right)-\int_{0}^{\infty }\frac{w\left(x,\theta \right){\tilde{h}}_{1}\left(x\right)}{A_{1}\left(x,\theta \right)}\;d\Pi \left(\theta \;|\;X_{1}^{**}\leq x\right)\\ \; & \;\;\\ \; & \geq {\tilde{h}}_{1}\left(x\right)\;\int_{0}^{\infty }\frac{w(x,\theta )}{A_{1}\left(x,\theta \right)}\;d\left[\Pi \left(\theta \;|\;X_{2}^{**}\leq x\right)-\Pi \left(\theta \;|\;X_{1}^{**}\leq x\right)\right]. \end{array}$$

It can be seen that $w\left(x,\theta \right)/A_{1}\left(x,\theta \right)$ is non-decreasing in θ∈χ. From assumption,

$$\int_{T}^{\infty }d\left[\Pi \left(\theta \;|\;X_{2}^{**}\leq x\right)-\Pi \left(\theta \;|\;X_{1}^{**}\leq x\right)\right]\geq 0,$$

for all T≥0. Lemma 7.1(a) of Barlow and Proschan [30] is applicable in (15) and provides the proof. □

### 4.3. A Link to Information Theory

The concept of entropy in information theory has played a prominent role in a broad area of science including statistical thermodynamics, urban and regional planning, business, economics, finance, operations research, queueing theory, spectral analysis, image reconstruction, biology and manufacturing (see, for example, El Gamal and Kim [35], Brillouin [36], Khinchin [37] and Grant [38]).

Stochastic comparisons of distributions have found a link to information theory in the literature (see, for instance, Ebrahimi et al. [39], Belzunce et al. [40], Nanda and Prasanta [41], Qiu [42], Toomaj and Di Crescenzo [43] and Toomaj and Di Crescenzo [44]).

Here before closing the paper, we impose a stochastic ordering property that leads to ordering of entropies of weighted distributions with weight functions given in Section 3.2. The extension of the Shannon entropy from the discrete case to the absolutely continuous case when dealing with lifetime random variables is defined by

$$H\left(X\right)=-\int_{0}^{\infty }f\left(x\right)\log\left(f\left(x\right)\right)\;dx,$$

where *f* is the pdf of non-negative random variable *X* with an absolutely continuous distribution function. Note that log, with convention 0log(0)=0 stands for the natural logarithm.

However, it is found that the entropy is related to the concept of dispersion of (random) variables. Being aware of this certitude, it is useful to concentrate on dispersion measures of probability distributions as well as their related stochastic dispersion orderings.

Let us recall from Shaked and Shanthikumar [34] that *X* with the pdf *f* and the cdf *F* is less (or equal) than (with) *Y* with the pdf *g* and the cdf *G* in dispersive order (denoted by $X\leq_{disp}Y$) whenever

$$F^{-1}\left(\beta \right)-F^{-1}\left(\alpha \right)\leq G^{-1}\left(\beta \right)-G^{-1}\left(\alpha \right),\;\;\mathrm{for}\;\mathrm{all}\;0<\alpha \leq \beta <1.$$

It follows from (3.B.25) in Shaked and Shanthikumar [34] that

$$X\leq_{disp}Y\;⟹\;Var\left(X\right)\leq Var\left(Y\right).$$

In spirit of Theorem 3.B.20(a) and Theorem 3.B.20(b) in Shaked and Shanthikumar [34] if *X* or *Y* has an increasing hazard rate function, then

$$X\leq_{disp}Y\;⟹\;X\leq_{hr}Y,$$

and if *X* or *Y* has a decreasing hazard rate function, then

$$X\leq_{hr}Y\;⟹\;X\leq_{disp}Y.$$

In accordance with Corollary 4.4 in Bartoszewicz [45], if *X* or *Y* has a decreasing reversed hazard rate function, then

$$X\leq_{disp}Y\;⟹\;X\leq_{rh}Y,$$

and if *X* or *Y* has an increasing reversed hazard rate function, then

$$X\leq_{rh}Y\;⟹\;X\leq_{disp}Y.$$

If *X* and *Y* are two random variables with supports $S_{X}=\left(l_{X},u_{X}\right)$ and $S_{Y}=\left(l_{Y},u_{Y}\right)$, respectively, then according to Theorem 3.B.13(a) in Shaked and Shanthikumar [34] when $l_{X}=l_{Y}>-\infty ,$

$$X\leq_{disp}Y\;⟹\;X\leq_{st}Y,$$

and also according to Theorem 3.B.13(b) in Shaked and Shanthikumar [34] when $u_{X}=u_{Y}<\infty ,$

$$X\leq_{disp}Y\;⟹\;X\geq_{st}Y.$$

The weight functions $w_{\theta }\left(x\right)$ and $v_{\theta }\left(x\right)$ considered in the following theorem depend on *x* only through F(x) and G(x), respectively.

**Theorem** **9.** *Let $X_{w_{\theta }}$ and $Y_{v_{\theta }}$ be the weighted versions of X and Y with weight functions $w_{\theta }\left(x\right)=d_{\theta }\left(F\left(x\right)\right)$ and $v_{\theta }\left(x\right)=d_{\theta }\left(G\left(x\right)\right)$, respectively, where θ∈χ is a parameter and $d_{\theta }$ is a non-negative function so that $\int_{0}^{1}d_{\theta }\left(u\right)du<\infty$ for any θ∈χ. Then, $X\leq_{disp}Y$ implies $H\left(X_{w_{\theta }}\right)\leq H\left(Y_{v_{\theta }}\right)$ for all θ∈χ.*

**Proof.**  Take $b\left(\theta \right)=\int_{0}^{1}d_{\theta }\left(u\right)du$ and observe that, for all θ∈χ,

$$E\left(w_{\theta }\left(X\right)\right)=\int_{0}^{\infty }d_{\theta }\left(F\left(x\right)\right)f\left(x\right)\;dx=b\left(\theta \right)$$

and also

$$E\left(v_{\theta }\left(Y\right)\right)=\int_{0}^{\infty }d_{\theta }\left(G\left(y\right)\right)g\left(y\right)\;dy=b\left(\theta \right).$$

Note that

$$\begin{array}{cc} H(X_{w_{\theta }}) & =-\int_{0}^{\infty }\log\left(\frac{d_{\theta }\left(F\left(x\right)\right)f\left(x\right)}{b(\theta )}\right)\frac{d_{\theta }\left(F\left(x\right)\right)f\left(x\right)}{b(\theta )}\;dx\\ \; & =-\int_{0}^{\infty }\log\left(\frac{d_{\theta }\left(F\left(x\right)\right)}{b(\theta )}\right)\frac{d_{\theta }\left(F\left(x\right)\right)}{b(\theta )}f\left(x\right)\;dx-\frac{1}{b(\theta )}\int_{0}^{\infty }d_{\theta }\left(F\left(x\right)\right)f\left(x\right)\log\left(f\left(x\right)\right)\;dx\\ \; & =c\left(\theta \right)-\frac{1}{b(\theta )}\int_{0}^{1}d_{\theta }\left(u\right)\log\left(f\left(F^{-1}\left(u\right)\right)\right)\;du, \end{array}$$

where $c\left(\theta \right)=-\int_{0}^{1}\left(d_{\theta }\left(u\right)/b\left(\theta \right)\right)\log\left(d_{\theta }\left(u\right)/b\left(\theta \right)\right)\;du$ is obviously independent of the underlying distribution. In a similar manner,

$$H\left(Y_{v_{\theta }}\right)=c\left(\theta \right)-\frac{1}{b(\theta )}\int_{0}^{1}d_{\theta }\left(u\right)\log\left(g\left(G^{-1}\left(u\right)\right)\right)\;du.$$

From (3.B.23) in Shaked and Shanthikumar [34], $X\leq_{disp}Y$ implies that $f\left(F^{-1}\left(u\right)\right)\geq g\left(G^{-1}\left(u\right)\right),$ for all u∈(0,1). Thus, we conclude that

$$\log\left(\frac{f(F^{-1}\left(u\right))}{g(G^{-1}\left(u\right))}\right)\geq 0,\;\mathrm{for}\;\mathrm{all}\;u\in \left(0,1\right).$$

As a result, for all θ∈χ, we deduce $H\left(Y_{v_{\theta }}\right)-H\left(X_{w_{\theta }}\right)=\frac{1}{b(\theta )}\left(\int_{0}^{1}d_{\theta }\left(u\right)\log\left(\frac{f(F^{-1}\left(u\right))}{g(G^{-1}\left(u\right))}\right)\;du\right)\geq 0.$ □
